# Innovative Drug Delivery Strategies for Enhanced Treatment of Antimicrobial Resistance 

**DOI:** 10.2174/0115672018415077251201092315

**Published:** 2026-06-02

**Authors:** Pranay Wal, Ankita Wal, Ajay Kumar, Hanish Singh Jayasingh Chellammal, Pratap Kumar Patra, Manish R. Bhise, Dileep Kumar, Sanjana Gupta, Amin Gasmi

**Affiliations:** 1 Department of Pharmaceutical Sciences PSIT-Pranveer Singh Institute of Technology (Pharmacy), NH-19 Kanpur Agra Highway, Bhauti Kanpur, UP, India;; 2 Department of Pharmacology, Sardar Patel College of Pharmacy, Gorakhpur, U.P., 273013, India;; 3 Department of Pharmacology and Life Sciences, Faculty of Pharmacy, Universiti Teknologi MARA (UiTM), Puncak Alam, 42300, Selangor, Malaysia;; 4 School of Pharmacy & Life Sciences, Centurion University of Technology & Management, Bhubaneswar, 752050, Odisha, India;; 5 Department of Pharmaceutics, SGSPS, Institute of Pharmacy, Akola, Affiliated to SGBAU, Amravati (MS) India;; 6 Department of Pharmaceutical Chemistry, Manipal College of Pharmaceutical Sciences, Manipal Academy of Higher Education, Manipal, Karnataka, 576104, India;; 7 Department of Pharmaceutics, Sardar Patel College of Pharmacy, Gorakhpur, U.P., 273013, India;; 8 Société Francophone de Nutrithérapie et de Nutrigénétique Appliquée, Villeurbanne, France;; 9 International Institute of Nutrition and Micronutrition Sciences, Saint-Étienne, France

**Keywords:** Antimicrobial resistance, nanomedicine, novel drug delivery systems, liposomes, nanoemulsions, polymeric nanoparticles

## Abstract

**Introduction:**

Antimicrobial resistance (AMR) presents a major global health threat, driven by the overuse and misuse of antibiotics. Traditional drug delivery systems face significant limitations in effectively addressing this issue, including poor targeting, low bioavailability, and the development of resistance. Nanoparticle-based drug delivery systems (NDDS) have emerged as a novel approach to overcome these limitations and combat resistant bacteria by improving drug delivery and targeting. This review aims to evaluate the mechanisms of AMR and the effectiveness of nanoparticle-based drug delivery systems in combating resistant bacteria.

**Methods:**

The methodology employed in this review involved a systematic search of peer-reviewed literature in databases such as PubMed, Google Scholar, Scopus, and Web of Science. The search strategy focused on identifying relevant articles using keywords and phrases such as "nanoparticle drug delivery systems," "antimicrobial resistance," "nanomedicine," "liposomes," "nanoemulsions," and "polymeric nanoparticles." The review included studies evaluating the mechanisms of antimicrobial resistance, the applications of NDDS, and their implications and advancements in combating AMR.

**Results:**

Nanoscale drug delivery vehicles and antibacterial nanoparticles have demonstrated significant potential as effective treatments for infections. Nanoparticles, with their small and controllable size, offer unique properties such as enhanced reactivity, a functionalizable structure, and a high surface area-to-volume ratio. NDDS, including liposomes, nanoemulsions, solid lipid nanoparticles, polymeric nanoparticles, and metal nanoparticles, have gained attention due to their ability to enhance drug bioavailability and targeting effectiveness.

**Conclusion:**

Nanoparticles present a promising strategy to combat AMR. Their capacity to bypass traditional resistance mechanisms and improve drug delivery underscores their potential in the development of effective antimicrobial therapies. Further research is essential to optimize nanoparticle formulations for clinical translation, ensuring their safety and efficacy in treating resistant bacterial infections. This review highlights the importance of integrating nanotechnology into antimicrobial strategies to address the growing challenge of AMR.

## 1 INTRODUCTION

Bacterial infections pose a significant threat to human health, with AMR contributing to increased morbidity and mortality globally. It is estimated that superbug infections cause more than 700,000 deaths worldwide annually [1]. Antibacterial medications are essential for treating bacterial infections. Still, microbes have grown more resistant to antimicrobial agents as a result of the overuse of these drugs in medical, farming, and animal production settings. This can result in a number of bacterial diseases and pose an important risk to human wellness and life. The ability of a microbe to evade the antimicrobial drug's mode of action and hence live is known as resistance to antibiotics (Fig. **1**) [2]. Most ATBs must, in theory, travel through the microbe's cell membrane before reaching their target region. Consequently, ATB can cause biochemical disruptions in cells, which may end in the inhibition of cell development (bacteriostatic medications) or death of cells (bactericidal drugs) [3].

Mechanisms of AMR include enzymatic degradation of antibiotics, modification of antibiotic targets, activation of efflux pumps, and biofilm formation. Each component illustrates a distinct bacterial survival strategy contributing to treatment failure (Fig. **1**).

Pathogens, nonetheless, have quickly evolved a number of strategies to avoid the harmful effects of ATBs. Frequently mentioned resistance mechanisms include, but are not limited to, altering and protecting the ATB targets, inactivating ATB medications, and limiting the ATB's ability to enter its target site by limiting entry or enhancing efflux. Drug-resistant microbes can therefore lead to larger drug dosages, more toxic therapies, and prolonged hospital stays, which can have a detrimental impact on the community and medicine by increasing mortality [4].

Due to their intracellular targets, some antibiotics need to navigate the pore protein in the membrane's outer layer and the active transport channel in the middle layer to reach the bacterial cell. Once inside, the antibiotic attaches to its target region and interferes with the biochemical reactions of the bacteria, which either inhibits or kills the cell.

ATB compounds in the periplasm or cytoplasm can be destroyed or their chemical makeup altered *via* the creation of enzymes; alternate binding locations can be created to avoid vulnerable targets for therapy from attaching to the antibiotic; the arrangement and functioning of the target can be altered; or an intricate efflux pump can be developed or upgraded to swiftly pump the antibiotic out of bacterial cells. These are just a few of the evasion tactics that bacteria can adopt when they become resistant to antibiotics [5]. Antimicrobial resistance (AMR) is currently one of the biggest problems facing the world's healthcare and economy, despite attempts to produce novel molecular entities through investigation and development.

Thus, it has grown to be a global issue and jeopardised the efficacy of universally accessible basic infection treatment and preventive measures [6]. The primary course of action recommended by the World Health Organization (WHO) and government health authorities has traditionally involved limiting the application of antibiotics while enhancing sanitation and antibiotic disposal procedures. Thus, to safeguard and fortify public health measures against the issue of antibiotic resistance globally, we require increased international collaboration and interdisciplinary research initiatives. Nevertheless, the process of creating novel adjuvant medicines, biological products, or antibiotics is not only challenging, but it is also unable to keep pace with the rate at which drug resistance is increasing. Therefore, novel antimicrobial treatments with clinical approval are desperately needed [7].

Because they are not site-specific and merely use one antimicrobial process, present individual therapies using nanoparticles (NPs) or antibiotics have a greater likelihood of bacteria resistance emerging. Conversely, the combination therapy of antibiotics and nanomaterials uses many antimicrobial approaches to target a specific target [8]. Moreover, because nanoparticles may mitigate antibiotic change in the human system, minimize antibiotic levels and quantity in the body, and avoid interactions of drugs with other microbes from the injected host, which might raise the potential of establishing antibiotic resistance, they can be useful instruments for coating or tethering antimicrobial agents to combat microbial drug resistance [9].

Therefore, in order to design more secure and efficient nano drug delivery systems (NDDS) to avert antibiotic resistance, it is imperative to examine the effects of NDDS in both the natural world and mammalian cells. To find out such information, particular analytical techniques must be used to ascertain their movement, reaction, ecotoxicity, and persistence in species and the environment. This section will address the current understanding of the production and consequences of combinatory NPs and antibiotics. First, the various drug delivery methods that are currently in use will be discussed, along with their characteristics, production, and toxicity, as well as how they interact with individuals, animals, and the surroundings [10].

This review presents a novel and integrative perspective on the role of NDDS in addressing the global challenge of AMR. While several reviews have touched upon nanotechnology applications in medicine, this manuscript uniquely synthesizes current insights into how different types of nanoparticles-including liposomes, polymeric nanoparticles, nanoemulsions, nanogels, solid lipid nanoparticles, and metal-based nanoparticles-interact mechanistically with microbial structures and resistance pathways. It also elaborates on their role in circumventing traditional barriers such as biofilm formation, enzymatic degradation, and efflux pumps. By providing a comparative analysis of traditional versus nanoparticle-based delivery systems, this article fills a critical knowledge gap and highlights the translational potential of NDDS in clinical antimicrobial therapy. Furthermore, it incorporates up-to-date studies, patented technologies, and specific antimicrobial formulations, thereby offering an advanced resource for researchers and clinicians aiming to design next-generation antimicrobial interventions.

## 2 MECHANISMS OF ANTIMICROBIAL DRUG RESISTANCE

Treatment of infectious illnesses is significantly hampered by antimicrobial medication resistance. Microorganisms can become resistant to antimicrobial treatments through a variety of intricate ways. Bacterial resistance to antibiotics can occur through a number of mechanisms, such as the antibiotics' enzymatic degradation; the antibiotics' enzymatic modification of their cellular targets; Genetic mutations; and Efflux pumps, which lower the level of cellular concentrations [11].

### 2.1 Efflux Pumps

It is well recognised that efflux pumps have a significant impact on the development of resistance to antibiotics in food-borne pathogens. These transport proteins have a role in the expulsion of several hazardous substrates and antibiotic classes. Antibiotic-resistant bacteria that have been identified from humans as well as animals have increased over the past 20 years [12].

Numerous studies have been conducted on efflux-mediated resistance, which is frequently linked to the relationship among active drug export (via efflux pumps) and decreased drug uptake (mostly as a result of modifications in outer membrane accessibility) [13]. These efflux networks are present in both eukaryotic and Gram-positive bacteria, and they play a role in the identification and extrusion of harmful substances from cells. Because of the chemical makeup of the cell membrane, efflux-mediated resistance in Gram-negative bacteria is a more complicated issue [14]. Efflux pumps may be specific or broad-spectrum in substrate activity. Six to eighteen per cent of all identified transporters are recognized or suspected drug efflux transporters, according to an analysis of accessible bacterial genetic sequences [15]. The bacterial cell's incoming antibiotic molecule is recognized by the efflux pump, which then attaches itself to it. After that, the pump expels the antibiotic from the cell by using energy. The efflux pump blocks the antibiotic from killing or suppressing the bacteria by ejecting it from the bacteria before it can reach its intended location (Fig. **1**). Such export systems fall into one of two categories: pumps with one or more components. The periplasmic area is reached by single-component pumps when their substrates are transported through the cytoplasmic membrane. Multi-component pumps, on the other hand, exist in Gram-negative bacteria and are made up of three proteins: TolC, a tribarrel protein which lies adjacent to the transporter protein and creates a channel that traverses the cell envelop, allowing the substrate to get from the outside; a fusion protein that binds the transporter protein to the exterior of the lipid arm of the plasma membrane; as well as the carrier protein itself, which recognizes a substrate present in the periplasmic space or cytoplasm, underneath the inward collateral of the plasma membrane [16]. Such tripartite pumps are especially suited to work in tandem by lowering outer pores to provide drug resistance since they span the whole Gram-negative cell envelope. The membrane-associated fusion protein & the cytoplasmic membrane efflux pump are encoded by the two genes that make up this efflux mechanism, acrA and acrB, respectively. The AcrA (94%) and AcrB (97%) proteins of E. coli and the AcrA and AcrB proteins of S. enterica serovar Typhimurium exhibit significant levels of similarity [17]. But Salmonella possesses three fusion proteins, compared to two in E. Coli. Whenever the acrA gene is removed, additional fusion proteins satisfy the fusion protein's absolute need [18]. The minimum inhibitory concentration (MIC) of therapeutically important antibiotics is not significantly affected by the omission of either acrD or acrF from S. enterica serovar Typhimurium, according to several investigations. On the other hand, it has been demonstrated that the deletion of any of these genes increases the expression of AcrB; similarly, the deletion of AcrB increases the expression of AcrD or AcrF [19].

### 2.2 Biofilm Formation

Pathogenic bacteria have been the subject of microbiological studies that have concentrated on the analysis of bacteria that develop in a liquid medium and their capacity to cause illness. However, more recently, the significance of bacteria thriving on solid surfaces has been acknowledged, and extensive studies have been conducted in this area [20]. An assembly of microorganisms with the ability to stick to one another or to a solid surface is known as a biofilm framework. These adhering cells are often immersed in an extracellular polymeric matrix that they have generated on their own. Many different species of bacteria, along with other microorganisms, are frequently found in biofilms. This has been proposed that biofilms are the predominant mode of proliferation for bacteria in nature [21]. The fact that biofilms are present offers a steady supply of bacteria for infection, which is another significant effect of their existence. Changes in the global genetic control of bacteria are also linked to the development of a biofilm [22].

The process of developing biofilms is complex and requires a number of successive processes. While filamentous fungi need to create their biofilms through multiple extra steps, such as the creation of mature filaments and terminating biofilm morphology, bacteria and yeasts have comparable biofilm formation mechanisms [23]. Cell attachment is typically the first step in the production of biofilms, which are then followed by the development of microcolonies, biofilm growth, and ultimately dispersion. As long as such cells are treated with antibiotics, their initial surface adherence is reversible. Once the cells have attached themselves to the surfaces and started to multiply, further actions cannot be undone [24]. Biofilms are dynamic and adapt to environmental and microbial signals. AMR is a natural reaction that microorganisms have developed to withstand harsh conditions. A few factors that have led to that phenomenon are human carelessness in the unchecked prescription of antibiotics, the incapacity of some antibiotics—like aminoglycosides and beta-lactams—to enter cells, or the reduced retention capabilities of some antibiotics, like macrolides and fluoroquinolones [25].

### 2.3 Enzymatic Degradation

Enzymes are essential for the emergence of drug resistance because they facilitate a variety of enzymatic processes, including group transfer, hydrolysis, and redox mechanisms. Thanks to the development of many enzymes, antibiotics can now be targeted, their weak connections broken, and their action eliminated. A key mechanism in the creation of antibiotic resistance in bacteria is hydrolysis. One such mechanism is the action of beta-lactamases, an enzyme that hydrolyzes the 4-membered lactam ring of beta-lactam antibiotics, to produce a biologically inert compound [26]. This enzyme could be encoded chromosomally or on extrachromosomal components of the bacterium. Based on the similarity of the amino acid series, they are divided into four categories: A, B, C, and D [27].

Another group of antibiotics wherein resistance to medication develops is macrolide antibiotics, which are mainly used to treat respiratory infections. Bacterial esterase hydrolyzes the macro lactone ring of these medicines. The erythromycin esterases EreA and EreB catalyse the hydrolysis of macrolides like erythromycin. The foundation of contemporary antibacterial chemotherapy is comprised of antibiotics, specifically azithromycin and erythromycin [28].

The largest and varied class of enzymes that cause resistance are called group transferases. They work by covalently altering antibiotics, which impairs target affinity. A class of enzymes known as acyltransferases is responsible for catalyzing the substitution of the acetyl ring of acetyl-CoA to the amine or 3-hydroxyl groups on antibiotics, which inactivates the antibiotics [29].

The predominant non-protein thiol in cells is glutathione (GSH). Enzymes associated with GSH are the primary defence mechanism that shields cells from electrophiles. Such electrophiles are produced by radiation, chemotherapy, contact with certain environmental mutagens, or perspiration. It functions by limiting the effectiveness of many medications by safeguarding intracellular elements (particularly proteins and DNA) and making them more hydrophobic [29]. The FosA (thiol transferase) protein, a Mn (II)-dependent metalloenzyme expressed in the genetic material of Pseudomonas aeruginosa, catalyzes the incorporation of GSH to Fosfomycin, an organic substance that imparts a broad spectrum of antimicrobial abilities against microorganisms, but only in the presence of the monovalent cation K [30].

Antibiotics additionally affect the bacterial ribosome, both at the 30S ribosomal subunit's encoding location or within the 50S subunit's peptidyl-transferase portion (where they hinder the synthesis of proteins by either disrupting the attachment of aminoacylate tRNAs at the A-/P-sites or obstructing the movement of the developing polypeptide chain through the ribosomal passageway). One of the pathways of tolerance to aminoglycosides is the A-site methylation of 16S rRNA of the 30S subunit by bacterial 16S rRNA methyltransferases, which disrupts the antibiotic's capability to attach to the ribosome and hinders the aminoacyl-tRNA from exact attachment to the anticodon, resulting in an inaccuracy in protein synthesis and cell death [31].

## 3 TRADITIONAL ANTIBIOTIC DELIVERY SYSTEMS

The usual techniques for giving patients antibiotics are known as traditional antibiotic delivery systems. These techniques have been around for a while. Numerous restrictions apply to conventional antibiotic administration systems, potentially compromising their efficacy and safety. Below list of a few of the conventional antibiotic delivery methods along with some of their drawbacks [32-35].

## 4 NOVEL DRUG DELIVERY SYSTEMS

The creation of NDDS offers fresh approaches to illness prevention and treatment. As NDDS in pre-clinical activities and clinical studies, liposomes, polymeric micelles, nanogels, nanoparticles of inorganic material, and inorganic/organic (core/shell) nanoparticles have all been used extensively in recent years. By prolonging the duration of action, enhancing potency and aiming to eliminating drug resistance, and lowering immunogenicity and toxic effects, such systems can enhance the pharmacological and medicinal qualities of pharmaceuticals. These days, the experimental and clinical phases are using an increasing number of NDDS [36].

### 4.1 Nanoparticles' Influence on Microbial Resistance

The main justification for considering nanoparticles as an antibiotic substitute is their potential to successfully stop microbial drug resistance in specific circumstances. Antibiotic overuse has given rise to several health risks for the general public, including superbugs resistant to all current medications and drug-resistant epidemics [37]. In order to tackle medication resistance, it is important to find new, efficient bactericidal agents. NPs have been investigated and identified as a viable solution to this problem [38]. NPs, yet, occasionally have the potential to encourage the development of bacterial resistance [39]. A growing array of NP variations and NP-based materials has been employed as a novel defence mechanism against multidrug-resistant (MDR) and resistant microbes [40]. How different kinds of NPs battle microbial resistance varies. Nanomaterials as antibacterial complementary to antibiotics are extremely intriguing and have generated significant attention, as they could fill gaps wherein antibiotics frequently fail, which is one of the acknowledged links involving nanomaterials and antibacterial effects. Furthermore, by acting "as a good carrier," nanoparticles can enhance and support conventional antibiotics [41].

Most nanoparticles can block common resistance mechanisms like biofilm inhibition and membrane rupture. Because of their high surface area-to-mass ratio and nanoscale size (\~100 nm), they can interact with bacterial cells more effectively. Their strong bactericidal effects are a result of their special physicochemical characteristics, which set them apart from traditional antibiotics. The effectiveness of nanoparticles in combating microbial resistance has been shown in numerous instances. For example, silver nanoparticles (AgNPs) have inherent antibacterial qualities because they may break down bacterial cell walls, denature proteins, and produce reactive oxygen species (ROS), all of which harm biological components. It has been demonstrated that AgNPs work in concert with antibiotics like ampicillin and ciprofloxacin to increase their antibacterial efficacy against resistant strains of S. aureus and E. coli. Similarly, by releasing Zn2+ ions and causing oxidative stress, zinc oxide nanoparticles (ZnO NPs) disrupt bacterial metabolism and DNA replication, aiding in the neutralization of resistance mechanisms in Pseudomonas aeruginosa and Bacillus subtilis [42]. Liposomal formulations, such as liposomal amikacin and moxifloxacin, overcome resistance related to poor permeability and biofilm shielding by enhancing medication penetration into biofilms and maintaining therapeutic concentrations at infection sites. By avoiding bacterial defences, polymeric nanoparticles such as plectasin encapsulated in PLGA also show improved transport and less cytotoxicity, especially in intracellular infections. These illustrations highlight the adaptability and effectiveness of nanoparticles as synergistic carriers in combination therapies or as independent antimicrobial agents, offering a powerful defence against multidrug-resistant (MDR) infections [43].

### 4.2 NDDS vs Traditional Antibiotic Delivery Systems

The primary benefits of using nanoparticles as a carrier for antibiotic therapy over conventional distribution methods are, NPs' ultra-small and controlled size makes them ideal for fighting intracellular microbes and carrying out antibacterial activities [44]. Since antibiotics have limited membrane transfer, treating infections produced by intracellular bacteria and drug-resistant strains is challenging when using antibiotics. Therefore, average-sized drugs have minimal impact on intracellular microorganisms. To get around this restriction, a modified therapy plan employing drug-loaded NPs as middlemen has been put forth. Most NP types are so small that host phagocytes may readily phagocytose them [45].

Furthermore, the majority of drugs can be distributed intracellularly due to the flexibility of NPs' ability to get into host cells *via* endocytosis. This is because many different types of NPs have structures that make them ideal for carrying substances, like liposomal NPs, whose exteriors are made of a few bilayers of lipids encompassing sphere-shaped NPs. Antibiotic serum concentrations can be raised with the aid of NP carriers, which can also shield the medications against target bacterial resistance. Medications are shielded from harmful chemical responses within NP carriers, allowing for the maintenance of the medications' potency [46].

Another crucial function is defence against the target bacteria's resistance mechanisms. Numerous NPs have been demonstrated by researchers to circumvent those mechanisms,18 preventing medication resistance. For instance, dendrimers can prevent P-glycoprotein-mediated efflux in the gastrointestinal tract [47]. Antibiotics can be targeted to an infection location with the aid of NP carriers, reducing systemic adverse effects. Without a carrier, it is challenging to promote high-dose drug absorption at the intended site and avoid side effects when using traditional antibiotics. Antibacterial delivery techniques based on NPs minimize side effects by delivering the medication to the point of action. Since a greater quantity of antibiotics is administered to the infection site, their unwanted side effects are precisely weakened [48].

## 5 NOVEL APPROACHES TO ANTIBIOTIC DELIVERY USING NANOMATERIALS

The creation of innovative drug delivery techniques for the administration of antibiotics is a vital step in the battle against bacterial infections. Novel delivery approaches are being investigated by scientists in an effort to improve antibiotic efficacy, targeting, and safety. Fig. (**2**) illustrates these innovative methods that aim to enhance medication stability, offer regulated release, and guarantee that antibiotics are delivered to the infection site with greater efficiency.

### 5.1 Liposomes

Among the most promising NDDS utilized in the discipline of antimicrobial research is liposomes [49]. Liposomes are now extremely effective antibacterial DDS thanks to significant advancements in liposome development [50]. Liposomes are spheroid vesicles that are structurally made up of an aqueous centre encircled by one or more lipid bilayers, which are often made of phospholipids or other amphiphilic lipids. Every liposome's membrane is roughly 4 nm thick, and its sizes typically vary from 20 nm to tens of microns [51]. It was successfully discovered that liposomes can be efficiently charged with charges to produce cationic, anionic, and neutral liposomes. Endocytosis and fusion are the primary ways that liposomes and cells interact. Moreover, liposomes are easily combined with additional targeting macromolecules to efficiently deliver medications to the infection region [52].

More significantly, the FDA had approved multiple liposome preparations, including Arikayce liposome, for the treatment of Mycobacterium avium, or they had demonstrated excellent therapeutic efficacy in anti-infectious condition clinical trials. Liposomes can also evade the drug resistance pathway of bacteria because they produce hydrolytic enzymes and alter the permeability of their membranes. Once the liposomes have entered the bacterium, they may react with the lipoproteins and lipopolysaccharides of the membrane on the outside, fuse with the bacterial cell membrane, and deliver the antibacterial medications to stop the bacteria from growing [53].

While liposomes offer many benefits, they are not without drawbacks. These include low hydrophilic chemical capture effectiveness, significant solvent residue stages, micron size and difficulties adjusting particle size variation. To overcome these constraints, scientific research has suggested the SuperLip (Supercritical-Assisted Liposome Formation) supercritical-assisted procedure, which produces liposomes steadily over an annual period of time with solvent residues lesser than those allowed by the Food and Drug Administration in the United States, well-controlled particle size distribution, and controllable liposome manufacture at the nano- or micrometer-level. Antibiotic encapsulation has also been investigated using this method, with up to 99% efficiency for encapsulation [54].

Because liposomes have robust blood-brain or plasma membrane barriers, they offer consistent drug loading, longer pharmacokinetics, fewer off-target unwanted effects, and increased effectiveness in getting to disease regions. Currently, a wide range of liposome medications are either on the market or in the clinical trial phase. A substantial number of these medications are antibacterial. Examples include Amikacin Sulfate Liposome Injection, Amphotericin B Liposome for Injection, and Econazole Nitrate Liposome Gel. Liposomes are a sort of tailored preparation that has the most developed and earliest clinical usage as a drug carrier [55].

### 5.2 Polymeric Nanoparticles

Polymer micelles are self-assembling structures created in solution in water by amphiphilic block copolymers. These solid, round aggregates range in size from 10 to 100 nm and are made of hydrophobic and hydrophilic blocks in amphiphilic copolymers [56]. Amphiphilic polymer molecules in water-based solutions persist as individual molecules at low amounts, much like small-molecule surfactants. The hydrophobic areas of the polymer interact with one another to create micelles when the quantity present surpasses the critical micelle concentration (CMC) as a result of hydrophobic, electrostatic in nature bonds of hydrogen, along with various molecular factors. Owing to their chemical and biophysical characteristics, they have demonstrated considerable promise as a drug delivery mechanism [57]. Polymeric nanoparticles offer several benefits for antimicrobial drug delivery: (i) they maintain their structural integrity in physiological fluids and can withstand a variety of harsh preparation requirements; (ii) they can be easily tuned for specific properties, like size, zeta-potentials, along with drug release identities, by adjusting the polymer lengths, organic solvents, and surfactants utilized for NP the creation [58]. (iii) they can be easily and versatilely surface functionalized for combining medication and target ligands [59].

It has been shown that using polymeric NPs for transporting different antimicrobial medicines has significantly increased the effectiveness of therapy in curing a wide range of infectious disorders. For example, treating S. typhimurium infections with encapsulating ampicillin in polymeric NPs and L. monocytogenes infections inside mice peritoneal macrophages [60] were both successful. The restricted ingestion of unstable or insufficiently absorbed medications can be circumvented by employing polymeric NPs [61]. Additionally, PEGylation of NPs can lengthen the drug's half-life in blood and enhance mucoadhesive qualities by lowering phagocytosis [62]. For the purpose of delivering antimicrobial drugs, polymer NPs appear to be the most appropriate nanoparticle framework.

The use of compostable and bioorganic polymers in the administration of peptide-based medications is an intriguing possibility because of its benign nature, compatibility, and disintegration behavior. Antimicrobial peptide (AMP) administration through the creation of polymeric medicinal nanostructures may present a great technological opportunity to increase medication security and bioavailability. Because of the well-established and broad range of antibacterial activity of CS, which may have beneficial interactions with antimicrobial compounds, CS-based NPs (CSNPs) are especially intriguing. Furthermore, CS nanostructures had been thoroughly researched for pharmaceutical delivery due to their biological-compatible features; this is also the case for AMP delivery [63, 64].

### 5.3 Nanogel

Nanogels are hydrogels at the nanoscale that are defined by a polymer network made up of water-soluble or amphiphilic polymers that are kept together by physical or chemical connections. Nanogels are helpful NDDSs because of numerous benefits, including easy spreading in water, a hydrophilic and soft look, a high water content, the capacity to encapsulate many different biologically active substances, and good body compatibility. Nanogel fundamental components typically have a diameter of 500 nm to 500 nm. The diversity of effective sizes is crucial in preventing rapid renal disintegration, which is enough to prevent the reticuloendothelial system from being absorbed. Nanogels exhibit potential permeability and can readily pass the blood-brain barrier (BBB) because of their nanosized properties [65].

Pharmaceuticals can be enveloped in nanogels *via* hydrophobic bonds, hydrogen bonding, or salt creation in along with physical packaging. The nanogel's interface is hydrophilic, making it difficult for blood cells to attach to it and preventing macrophages from phagocytosing it [66, 67]. pharmaceuticals of diverse kinds, such as hydrophilic and hydrophobic pharmaceuticals, peptide and protein drugs, and nucleic acid drugs, can be encapsulated in nanogels with varying shapes and compositions. Nanogels are an intriguing technology because of their high encapsulation efficacy, strong stability, and environmental sensitivity. Furthermore, nanogels exhibit excellent biocompatibility, being biodegradable and other qualities, such as increasing the effectiveness of drug-loaded chemotherapy treatments on drug-sensitive and drug-resistant tumor cells and sensitizing antibiotic-resistant pathogens like bacteria [68].

Both the cytotoxicity and antibacterial activity of antimicrobial nanogels can be dramatically changed by adjusting their composition. Whenever the hydrophobic molecules of the gels linked against the lipid membrane, the antibacterial agent triclosan, which was encapsulated in the produced nanogels, succeeded in the form of bacteria [69]. The bonding amongst aliphatic chains and triclosan is lessened as a result of their intercalation and penetration on the bacterial cell membrane, making the expulsion of triclosan upon membrane opening simpler and more straightforward. As a result, triclosan's potent local concentration within bacterial sections considerably rose. Triclosan's therapeutic impact was greatly enhanced by a multitude of folds lowering the minimum bactericidal concentration, which led to a reduction in the number of concentrations needed without compromising its effectiveness. Additionally, it is anticipated that this nanogels technology may lessen the negative consequences of antibacterial medicines as well as prevent future bacterial resistance formation [70].

### 5.4 Solid Lipid Nanoparticles

Solid lipid nanoparticles (SLNs) are a type of NPs consisting of solid lipids sustained in a solution of water by an emulsifying layer [71]. Their ability to administer medications at a regulated and site-specific pace has made this sort of lipid-based vesicular framework popular. Surfactants are also used in emulsification to increase the durability of SLN [72]. These biopolymers can be synthetic or natural, and they work well for encapsulating lipophilic medications. Compared to isolated antitubercular medications, SLNs including antitubercular pharmaceuticals made using the microemulsion process demonstrated a twofold suppression of Mycobacterium marineum.

In addition to acting as delivery mechanisms for antibiotic drugs, some NPs within SLNs exhibit antibacterial properties. Utilizing NPs as a nano-drug delivery technology can enhance antibiotics' antibacterial efficacy. Antibiotics have trouble penetrating human cells, but because of their extremely small shape and size, NPs can get past the membranes of afflicted cells. Therefore, they are involved in the defence against intracellular infections like multidrug-resistant Staphylococcus (MRSA) [73].

### 5.5 Nanoemulsion

A colloid particulate nano system made of a combination of water, oil, and an appropriate stabilizing surfactant is called a nanoemulsion (NE), often referred to as a microemulsion [74]. Because they have larger surface areas and less large particles than traditional emulsions, nanoemulsions are usually more durable and permeable. NEs are made up of nanometer-sized droplets stabilized by emulsifiers. The NE's durability and solubility in aqueous solutions made them an efficient drug transport mechanism [75]. Because of their inherent antimicrobial qualities, capacity to boost drug solubility, stability, and bioavailability, capability aimed at specific organs and cells, capacity to target biofilms, and ability to overcome antibiotic resistance, NE were recognized as an exciting means of antimicrobial delivery. There are several ways for administering NE [76].

Given its inherent virulence, propensity to cause several infections that can be fatal, adaptability to various environmental circumstances, and ability to create biofilms, Staphylococcus is possibly the pathogen that should be of the highest priority. Several antibiotics (clindamycin, linezolid, doxycycline) have been shown to have better antibacterial properties towards Staphylococcus thanks to the preparation of innovative nano-biotic formulations by Mohamed *et al*. Antibiotics were tested for their cytotoxicity and antibacterial activity as nanoemulsions. The results of this research demonstrated the potential therapeutic benefits of antibiotics loaded in nanoemulsion, showing them to have lower cytotoxicity and greater antibacterial activity than traditional free antibiotics [77].

### 5.6 Metal and Inorganic Nanoparticles

Metal and metal oxides, such as ZnO, Fe, Au, Ag, and titanium oxide (TiO2), are examples of inorganic NPs [78]. Inorganic nanoparticles have been widely investigated for a range of medicinal uses, including the treatment of bacteria. Inorganic pharmaceuticals can gain from NDDSs in a similar way as organic medications by strengthening their pharmacokinetic attributes, like improved targeting, loading of drugs, and immune response evasion. Metal NPs exhibit antibacterial properties based on their size and shape. This is achieved by various mechanisms, such as binding and altering their role of polymers and generating free radicals *via* ROS [79].

Due to their ability to target several cell components, including the cell wall, DNA, membrane, and protein, metal NPs are regarded as promising candidates for antibacterial treatments. As a complement to antibiotics, green synthetic metallic and metal oxide nanoparticles are thought to be a viable way to target bacteria. The fields of biomedicine and nanotechnology have made considerable use of magnetic NPs. Because these NPs interfere with a thiol group in bacteria, they induce damage, which makes their antibacterial action intriguing. Biosynthesized magnetic nanoparticles were examined for their antibacterial activity against a range of drug-resistant bacteria, including E coli, Salmonella typhi, S. aureus, P. aeruginosa, and Pasteurella multocida [80]. The ability of magnetic NPs to inhibit S. aureus and E. coli growth in a concentration-dependent manner was validated by the Agar-diffusion method. Furthermore, in comparison to the conventional medications, some magnetic nanoparticles showed significant activity against all microorganisms [81].

Although AuNPs don't have (natural) antibacterial action, yet do strengthen the antibacterial qualities of loaded antibacterial medications. Medication-loaded AuNPs may cause bacterial cell metabolism to degrade by collapsing the membrane current of the bacterium, inhibiting the function of ATPase, and stopping ribosome adherence to tRNA [82]. Several bacterial infections in the bones, joints, the oral cavity, skin, gastrointestinal tract, urinary system, and respiratory tract are treated with the antibiotic ciprofloxacin (CIP) [83]. For bacteria that are resistant to many drugs, AgNp exhibit extremely potent antibacterial activity. AgNP-chitosan/ montmorillonite nanocomposite films have the potential to regulate the morphological properties of AgNPs in addition to their notable ability to inhibit B. subtilis and E. coli [84].

## 6 STUDIES AND APPLICATIONS OF NDDS AS ANTIMICROBIALS

The technological advancement of NDDS, their comprehensive characterization, and the assessment of their effectiveness in the delivery of antimicrobial medications have all been the subject of research [85]. Table **1** lists investigations into various NDDS as antimicrobials, and Table **2** provides an overview of the antibacterial uses of different NPs, including information on target bacteria, mechanism of action, and kind of disease formulation.

## 7 PATENTS REPORTS

The list of patents utilizing NDDS as antimicrobial agents is provided in the next section (Table **3**) [106-113].

## 8 LIMITATIONS OF NANOPARTICLES

Although there are many benefits to using nanoparticles for the delivery of drugs, it is essential to be aware of any potential disadvantages.

### 8.1 Toxicity

Depending on their surface characteristics, size, shape, and composition, certain nanoparticles may be poisonous. Particularly, metal nanoparticles have the potential to produce ions that are detrimental to cells. Polymeric nanoparticles have the potential to break down into hazardous substances. To reduce toxicity, careful material selection and surface modification are crucial [114].

### 8.2 Off-target Effects

It's possible that nanoparticles won't always be directed solely to the intended site of action, which could result in adverse reactions in healthy tissues. This may restrict the treatment window and cause unfavourable side effects.

### 8.3 Long-term Stability

It can be difficult for nanoparticles to remain stable in biological fluids. They may interact with proteins, agglomerate, or degrade them, changing their characteristics and decreasing their effectiveness.

### 8.4 Production and expansion

It can be difficult and costly to produce nanoparticles on a large scale with reliable quality and characteristics. This poses a problem for the broad clinical application of treatments based on nanoparticles.

### 8.5 Long-Term Health Implications

Research is still being done on the long-term health effects of nanoparticle exposure. Accumulation, following repeated dosing, certain nanoparticles may build up in organs, including the kidneys, liver, or spleen. More research is needed to properly understand the accumulation's long-term implications.

### 8.6 Immune Responses

Nanoparticles may cause allergic or inflammatory reactions by interacting with the immune system. The type of nanoparticle and the immunological condition of the individual can affect the immune response.

### 8.7 Genotoxicity and Carcinogenicity

Some nanoparticles may damage DNA or aid in the formation of cancer, which raises concerns. Long-term research is required to evaluate these dangers, even though the data are limited [115].

## 9 OVERCOMING AMR MECHANISMS AND CLINICAL IMPLICATIONS OF NDDS

One of the biggest risks to world health is AMR, which undermines the effectiveness of current antibiotics and causes longer sickness, higher mortality, and rising medical expenses. Enzymatic inactivation of antibiotics (*e.g*., β-lactamases), alteration of drug-binding targets (*e.g*., penicillin-binding proteins in MRSA), activation of efflux pumps (*e.g*., AcrAB-TolC in Gram-negative bacteria), decreased membrane permeability, and biofilm formation that prevents drug penetration are some of the various bacterial strategies that lead to the emergence of AMR. Traditional antibiotics are generally unable to circumvent these complex, multidimensional resistance pathways, underlining the urgent need for novel, focused therapeutic alternatives.

NDDS are at the forefront of tackling these difficulties through personalised drug encapsulation, targeted delivery, and barrier-penetrating capabilities.

For example, liposomes can fuse with bacterial membranes, allowing antibiotics to be delivered directly into the cytoplasm while avoiding resistance mechanisms like efflux pumps. Vancomycin's liposomal version has demonstrated improved intracellular uptake and anti-MRSA effectiveness. In resistant infections, SLNs have also shown promise in avoiding efflux systems and preserving antibiotic release, which keeps therapeutic medication levels stable.

Drugs are encapsulated and shielded against enzymatic breakdown by β-lactamases and other hydrolytic enzymes by polymeric nanoparticles (such as PLGA and chitosan-based systems) [116]. For instance, against resistant strains of E. coli, ceftriaxone-loaded PLGA nanoparticles have demonstrated improved stability and bioavailability. In a similar vein, erythromycin-loaded chitosan nanoparticles demonstrated improved mucosal penetration and effectiveness against *Klebsiella pneumoniae* and *Pseudomonas aeruginosa*, two bacteria that are resistant due to decreased membrane permeability and efflux [117].

Another major obstacle in persistent infections, such as those in diabetic foot ulcers, prosthetic joint infections, and cystic fibrosis lungs, is biofilm-mediated resistance. By means of mechanical penetration, matrix breakdown, or local ROS production, NDDS provide biofilm-disruptive properties. By enhancing penetration and rupturing the extracellular polymeric substance (EPS) matrix, nanoemulsions containing rifampicin or chlorhexidine have shown strong biofilm suppression. Nanogels can enter biofilms deeply and release medications in a regulated way because of their hydrophilic and malleable structure. Nanogels coated with daptomycin have demonstrated enhanced efficacy against Enterococcus faecalis biofilms.

Metallic nanoparticles damage DNA, produce ROS, bind thiol groups in bacterial enzymes, and interfere with membrane potential. Particularly, AgNPs have demonstrated broad-spectrum effectiveness against infections that are resistant to many drugs, such as K. pneumoniae, P. aeruginosa, and Acinetobacter baumannii. By increasing absorption and upsetting cellular homeostasis, gold nanoparticles coupled with ampicillin or vancomycin have re-sensitised resistant strains of Staphylococcus aureus. Targeted delivery to internal bacterial reservoirs—a major location of resistance and persistence, particularly in illnesses like listeriosis and tuberculosis—is also made possible by NDDS. The uptake of anti-TB medications such as isoniazid and rifampicin within infected host cells has been enhanced using mannose-coated nanoparticles, which are intended for macrophage targeting. In a similar vein, dendrimer-based technologies enable accurate administration into pathogen-containing phagocytic cells. NDDS improve formulation flexibility in drug development and allows for the repositioning of already-approved antibiotics. For instance, when repackaged in nanocarriers, earlier medications such as colistin and rifabutin have regained their therapeutic efficacy. To treat systemic or central nervous system infections, NDDS also make it easier to transport medication across biological barriers like the gastrointestinal tract or BBB [118].

Approved products and continuing clinical trials demonstrate NDDS's translational potential. These platforms offer scalable, repeatable, and patient-compatible solutions, bridging the gap between preclinical discovery and bedside use. In the post-antibiotic era, NDDS enhance precision medicine approaches to infection therapy, slows the rise of resistance, and eventually improves global health outcomes.

## CONCLUSION

The development of sophisticated drug delivery techniques that improve the effectiveness of currently available antimicrobials and get beyond known resistance mechanisms has attracted a lot of attention due to the global problem of AMR. NDDS have a number of benefits over traditional formulations, especially those based on nanotechnology, such as liposomes, SLNs, nanogels, polymeric nanoparticles, and metallic nanoparticles. Numerous preclinical and early translational investigations have shown that they include enhanced drug solubility, targeted distribution, prolonged release, and decreased toxicity.

Major AMR mechanisms, such as enzymatic degradation, restricted membrane permeability, efflux pump activity, and biofilm-associated resistance, may be addressed by NDDS. These platforms may aid in maintaining or boosting the efficacy of current antimicrobials by improving intracellular penetration, safeguarding active therapeutic components, and encouraging site-specific distribution. Furthermore, in lab models, the combination of antibiotics with nanoparticle systems has demonstrated synergistic effects against bacteria that are resistant to multiple drugs.

Many NDDS are still at the experimental or investigational stage, even if some, like liposomal versions of amphotericin B and amikacin, have advanced into clinical trials or obtained regulatory approval. To evaluate their pharmacokinetics, safety, scalability, and clinical effectiveness in human populations, more research is required. Antimicrobial therapy that incorporates NDDS has the potential to improve treatment results, slow the emergence of resistance, and increase precision-based infection control.

All things considered, NDDS mark a significant advancement in the hunt for long-term and efficient methods to control antibiotic resistance. To guarantee patient safety and therapeutic benefit, their clinical translation must be carefully assessed through reliable trials and regulatory processes.

## Figures and Tables

**Fig. (1):**
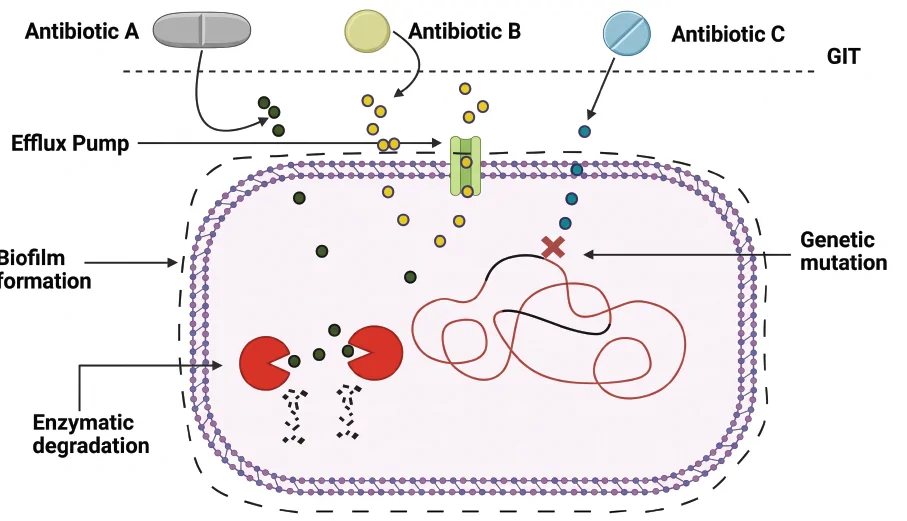
Antibiotic resistance by microbes.

**Fig. (2):**
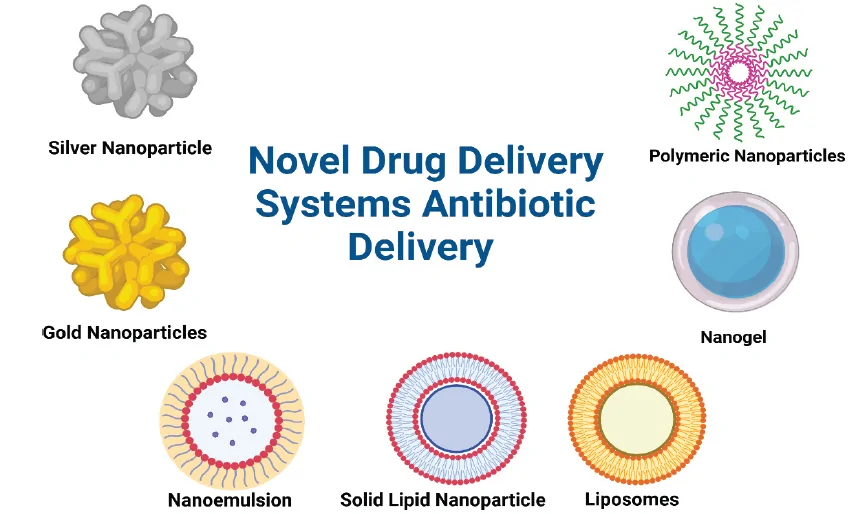
Novel drug delivery methods employed for antibiotic delivery.

**Table 1:** Antimicrobials NDDS formulations and their effects.

**S. No**	**Nanomaterials**	**Drug**	**Observation**	**Inference**	**References**
1.	Liposome	Moxifloxacin	The thin-film hydration approach was used to create the liposomal moxifloxacin formulation. The liposomes were between 50 and 70 nm in size. The free drug, neutral, and charged moxifloxacin liposomes had MIC concentrations of 10, 5, and 2.5 towards Pseudomonas aeruginosa, *etc*. The free drug, neutral, and cationic moxifloxacin liposomes have MICs of 1, 1, and 0.5 towards Staphylococcus aureus, respectively.	Moxifloxacin encapsulation in liposomes, particularly cationic vesicles, may improve their antibacterial qualities.	[86]
		Amikacin	The study investigated the biofilm access, drug release process, and *in vivo* antibacterial activity of an exclusive nanoscale liposomal formulation intended for inhalation administration of amikacin. Sputum or supernatants from Pseudomonas biofilms mediated the expulsion of amikacin *via* liposomes. When inhaled, liposomal amikacin was orders of magnitude more effective than inhaled free amikacin in diseased lungs, while being released slowly and continuously in healthy rat lungs.	The reason behind the higher *in vivo* effectiveness of inhaled liposomal amikacin in comparison to free medication in a 14-day infectious model can be attributed to the entry of biofilm with the targeted, sustained release from liposomes. Liposomal amikacin inhaled may be a significant treatment for persistent lung infections.	[86]
2.	Polymeric Nanoparticles	Plectasin	Phenotypic-loaded NPs' antibacterial activity was examined in bronchial epithelial Calu-3 cell monolayers harbouring S. aureus infection. At the concentrations tested, eukaryotic cell survival was not impacted by the plectasin-loaded NPs, but they did show increased efficacy when contrasted with non-encapsulated plectasin. In THP-1 macrophages and Calu-3 epithelial cells, the NPs were dispersed intracellularly in punctate patterns; however, A549 epithelial cells did not exhibit a discernible absorption of the NPs.	It seems that encasing plectasin in PLGA-based NPs is a workable way to increase plectasin's effectiveness towards infections in epithelial tissues.	[87]
3.	Nanogel	Triclosan	Loaded into the hydrophobic regions of the nanogel is triclosan. *via* the ruptured membrane, the hydrophobic antibiotic Triclosan can be effectively administered into the cell. By using this method, the effective amount of Triclosan at the bacterial site is significantly increased. In comparison to free Triclosan, the minimal inhibitory and bactericidal concentrations towards the Gram-positive bacteria S. aureus and S. epidermidis dropped three orders of scale.	The combined effects of physical devastation and active nanoinjection greatly increase the antimicrobial agents efficacy. The system for administering hydrophobic drugs maintains great potential for numerous additional uses, including the fight against AMR.	[88]
		Azithromycin	Delivery approaches were able to enter the bacterial biofilm, but the small poly(lactic-co-glycolic acid) NPs stayed in the biofilm more time compared to the hydrophilic hyaluronic acid nanogels; the Azithromycin nanogels demonstrated better cell specificity, alongside no apparent cellular damage for liver cells and lung epithelial cells; delivery techniques enhanced the anti-microbial and Anti virulence properties of azithromycin, in addition to enabled mitigation and elimination of preformed biofilms at significantly smaller amounts of the medication than non-formulated azithromycin.	While the hyaluronic acid nanogels demonstrated decreased mucin interactions along with enhanced decrease in pre-formed biofilms while retaining the low eukaryotic cell cellular damage of azithromycin, the NPs significantly increased the antimicrobial capacity and the biofilm avoidance efficiency of azithromycin.	[89]
4.	Solid Lipid Nanoparticles	Rifampicin	Using a modified microemulsion/sonication technique, rifampicin-loaded SLN were produced and evaluated. The mean size was around 319.7 nm, the PI was approximately 0.20, and the zeta potential was approximately 18.4 mV. The spherical form and encapsulating efficiencies were equivalent to 95.78 and 34.2%, respectively. Five days passed during the drug release. When compared to free Rifampicin, its antibacterial effect in bacterial and cell culture media was statistically significant (p<.05).	In summary, SLN appear to be a viable delivery method for augmenting Rifampicin's antimicrobial activity towards Brucella abortus.	[90]
5.	Nanoemulsion	*Thymus daenensis* essential oil	The essential oil's antibacterial property was evaluated towards the significant food-borne infection bacterium E. coli in both its pure and nanoemulsion forms. By assessing the MIC and MBC, antibacterial capacity was ascertained. Once the essential oil was transformed into a nanoemulsion, its antibacterial effect towards E. coli was significantly increased. This was explained by the essential oils' greater access to the bacterial cells. A 1-log decrease was seen for pure essential oil, but the nanoemulsion killed every bacterium in roughly 5 minutes, according to the analysis of the kinetics of microbial inactivation.	By strengthening their capacity to compromise the structural stability of cell membranes, the nanoemulsion seems to enhance the antibacterial properties of essential oils against E. coli.	[91]
6.	Metal and Inorganic Nanoparticles	Ampicillin	Polymersomes with ampicillin in the aqueous segment and AgNPs implanted in the hydrophobic segment were created. The spread of E. coli modified with a gene for ampicillin resistance (bla) was shown to be suppressed by these silver nanoparticle-embedded polymersomes (AgPs) in a dose-dependent manner for the first time. The growth of bacteria was unaffected by free ampicillin, AgPs lacking ampicillin, and ampicillin polymersomes lacking AgNPs. At a silver-to-ampicillin ratio of 1:1.64, the synergistic interaction among AgPs and ampicillin resulted in total growth suppression.	Antibiotic-resistant illnesses could be effectively treated with polymersome nanocarriers embedded with AgNPs.	[92]
7.		Plant peptide MBP-1	MIC and MBC of MBP-1 and AgNPs towards S. aureus were ascertained. In a rodent model, the combined antibacterial efficacy of AgNPs and MBP-1 was studied on S. aureus-caused infected wounds. It was discovered that MBP-1's MIC and MBC were, respectively, 0.6 and 0.7 mg/mL. AgNPs were shown to have a MIC and MBC of 6.25 and 12.5 mg/L, respectively. AgNPs and MBP-1 combination's MIC and MBC were determined to be 3.125 mg/mL and 0.5 mg/L, respectively, and 6.25 mg/mL and 0.6 mg/L. Following the application of MBP-1 and AgNPs together, the infected wound healed appropriately.	A mixture of MBP-1 and AgNPs was discovered to have an additive impact on the recovery of S. aureus-caused wounds with infection in a mouse model.	[93]

**Table 2:** Summarizing the antibacterial applications of various NPs, including their mechanism of action, target bacteria, and disease formulation type.

**S. No.**	**Nanoparticle**	**Mechanism of Action**	**Target Bacteria**	**Disease**	**Formulation Type**	**References**
1.	Silver (Ag) NPs	Disrupts cell membrane, generates reactive oxygen species (ROS)	E. coli, S. aureus, P. aeruginosa	Wound infections	Topical ointment, coating	[94, 95]
2.	Gold (Au) NPs	Penetrates cell walls, binds to sulfur-containing proteins, and generates ROS	E. coli, S. aureus, Salmonella typhi	Foodborne illnesses	Coating, suspension	[96, 97]
3.	Zinc Oxide (ZnO) NPs	Generates ROS, disrupts the bacterial cell membrane, and inhibits enzyme activity	E. coli, S. aureus, Bacillus subtilis	Skin infections	Cream, suspension	[98, 99]
4.	Titanium Dioxide (TiO2) NPs	Photocatalytic generation of ROS damages the cell membrane	E. coli, S. aureus	Surface infections, water disinfection	Coating, suspension	[100]
5.	Copper (Cu) NPs	Disrupts cell membrane, generates ROS, interferes with protein synthesis	E. coli, S. aureus, P. aeruginosa	Respiratory infections	Suspension, coating	[101, 102]
6.	Iron Oxide (Fe3O4) NPs	Generates ROS, disrupts the cell membrane, binds to and inhibits enzymes	E. coli, S. aureus	Wound infections	Topical gel, suspension	[103]
7.	Chitosan NPs	Disrupts the cell membrane, binds to DNA and inhibits replication	E. coli, S. aureus, P. aeruginosa	Gastrointestinal infections	Suspension, coating	[104, 105]

**Table 3:** List of Patents including NDDS as antimicrobial agents.

**S. No.**	**Patent No.**	**Title**	**Description**	**References**
1.	US8927024B1	Antimicrobial polyanhydride nanoparticles	The innovation offers materials and procedures for managing microbial infections in animals, stopping bacteria-infected cells from multiplying, and eliminate pathogens within infected cells. One way of treating an animal in require of this kind of care is to give it a composition made of polyanhydride microparticles, or NPs, which encapsulate multiple antimicrobial substances in a potent antibacterial amount. The antimicrobial chemicals are released when the microparticles, also known as NPs, break down by surface erosion over time. This process kills or inhibits the microorganisms and treats the illness. The NPs can concentrate in infected monocytes, dendritic cells, or both on or in other infected cells.	[106]
2.	US10953039B2	Utilizing polymers and antibiotics to enhance antimicrobial activity and inhibit antibiotic resistance	Methods for using combination therapy to treat any number of microorganism illnesses are given. For instance, one or more of the embodiments discussed herein may include a method that consists of using combination therapy to increase an antibiotic's antimicrobial activity. The antibiotic and a polycarbonate polymer functionalized with a guanidinium functional group may be used in a combination therapy.	[107]
3.	US10632070B2	Hydrogel toxin-absorbing or binding nanoparticles	Compositions containing a polymeric hydrogel loaded with a toxin-absorbing or binding NPs are made possible by the current invention. The foregoing compositions may also be used, according to this invention, to cure or avert a subject's contamination by a microbe that creates toxins, or to lessen or neutralize the effects of toxins. A biological toxin, such as one that is bacterial, fungal, viral, plant, or animal-based, is the model toxin.	[108]
4.	WO2016153979A1	Modulating antibacterial immunity *via* bacterial membrane-coated nanoparticles	Some NPs have an outside surface made of a bacterial membrane, such as one that is formed from a bacterial exterior vesicle, and an interior core made of a non-cellular substance. Formulations including the current NPs, such as pharmaceutical formulations and medication delivery methods, are also supplied. Uses of the current pharmaceutical pieces, systems for drug delivery, and NPs for curing and/or avoiding an illness or disorder, such as an infection by bacteria in a person, are also presented.	[109]
5.	WO2016205009A1	Treating infection by a platelet-targeting microbe using nanoparticles	Using, among other things, a sufficient quantity of NPs with an outer layer made of a cell membrane obtained from a platelet and an interior portion made of a non-cellular substance, the presented innovation offers techniques, combinations, and pharmaceutical compositions for averting and/or managing infection by a platelet-targeting microbe in a subject.	[110]
6.	US10285952B2	Nanoparticles leverage biological membranes to target pathogens for disease treatment and diagnosis	Procedures, combinations, and medicinal product compositions for addressing or avoiding infections in subjects using nanoparticles with two main components are described: (a) an outer surface with a cell membrane that is designed to cling to a pathogen which triggers the infection, and (b) a core within made of a non-cellular material. An infection brought on by a virus, bacteria, fungi or protozoa is considered an exemplary disease.	[111]
7.	CN110269847A	A kind of bionic nano material and the preparation method, and application thereof for neutralising bacteriotoxin	This invention describes a type of bionic nanotechnology, an approach for preparing it, and a usage for neutralizing bacteriotoxin. The bionic nanomaterial has good hydrophily and biological compatibility, making it effective at removing bacteriotoxin. It is anticipated that this will result in a secure and successful nano eye drop that neutralizes intraocular toxins. Bacteriotoxin reduces retinal tissue damage in entophthamia, and at low levels, the toxin's cell-damaging assess can be decreased by no less than one-third when using the bionic nanomaterial.	[112]
8.	US20210038772A1	Anti-infective antimicrobial-containing biomaterials	A biomaterial that has silver metal generated inside it and several fatty acid chains that are cross-linked simultaneously. Provided are the techniques for making the material. A number of anti-infective compounds can be released and delivered locally using the silver-containing biological material either by itself or in conjunction with a medical device.	[113]

## LIST OF ABBREVIATIONS

AMR: Antimicrobial Resistance
ATB: Antibiotics
BBB: Blood-Brain Barrier
CMC: Critical Micelle Concentration
GSH: Glutathione
MIC: Minimum Inhibitory Concentration
NDDS: Novel Drug Delivery Systems
NE: Nanoemulsion
NPs: Nanoparticles
SLNs: Solid Lipid Nanoparticles
WHO: World Health Organization
